# Persistent Burden of Schistosomiasis in South Africa: A National Laboratory-Based Analysis, 2019–2024

**DOI:** 10.3390/tropicalmed11060154

**Published:** 2026-06-05

**Authors:** Charlotte Sriruttan-Nel, Bhavani Moodley, John Frean, Dumisani Mlotshwa, Veerle Msimang

**Affiliations:** 1 Centre for Emerging Zoonotic and Parasitic Diseases, National Institute for Communicable Diseases, Johannesburg 2192, South Africa; johnf@nicd.ac.za (J.F.); veerlem@nicd.ac.za (V.M.); 2 School of Pathology, Faculty of Health Sciences, University of the Witwatersrand, Johannesburg 2050, South Africa; 3 Wits Research Institute for Malaria, Faculty of Health Sciences, University of the Witwatersrand, Johannesburg 2050, South Africa; 4 Department of Information Technology and Surveillance Data Warehouse, National Institute for Communicable Diseases, Johannesburg 2192, South Africa; dumisanim@nicd.ac.za

**Keywords:** schistosomiasis, bilharzia, neglected tropical disease, *Schistosoma*, parasite

## Abstract

Schistosomiasis remains the second leading cause of death among parasitic diseases globally, contributing substantially to chronic morbidity and disability. South Africa (SA) is endemic for schistosomiasis, with ongoing efforts to expand mass drug administration. In the absence of true prevalence data, this study retrospectively analysed public sector laboratory-confirmed schistosomiasis cases from 2019 to 2024. Over this period, 73,680 cases were microscopically diagnosed, with *Schistosoma haematobium* accounting for 99.9% of infections. The test positivity rate of 20 per 100,000 population is lower than previously reported, with the burden concentrated among boys aged 5–19 years, particularly in the KwaZulu-Natal, Limpopo, and Mpumalanga provinces. These findings highlight a persistent burden within defined demographic groups and geographic areas, while also suggesting possible early signs of improvement that are potentially linked to public health interventions. The results provide valuable evidence to inform the scale-up of national schistosomiasis control programmes and the prioritisation of interventions towards the most affected populations.

## 1. Introduction

### 1.1. Schistosomiasis: Global, Regional, and South African Context

Schistosomiasis (bilharzia) is a preventable and treatable parasitic disease. Despite this, millions of people across 78 countries still face morbidity and mortality related to the infection [1]. Poor communities in sub-Saharan Africa bear over 90% of the global disease burden, although the disease remains an important public health problem in parts of South America, the Middle East, and Asia [1]. According to the World Health Organization (WHO), only 30% (75.3 million) of the estimated 251.4 million people requiring preventive chemotherapy actually received treatment in recent years [1].

Among parasitic diseases, schistosomiasis is widely recognised as one of the most important neglected tropical diseases (NTDs), contributing to chronic morbidity, disability and long-term complications such as hepatic fibrosis, bladder pathology and increased cancer risk [2,3]. It ranks second only to malaria as a global cause of death, with an estimated 280,000 deaths annually from chronic complications [3,4,5]. Two decades ago, the WHO published the first list of NTDs, highlighting conditions that had been largely absent from the global health agenda [6]. The inclusion of schistosomiasis and other NTDs was justified by a growing body of evidence that their control directly contributes to achieving both the Millennium Development Goals and, later, the UN Sustainable Development Goals [6].

Control of schistosomiasis has been prioritised within global health agendas, including the WHO roadmap for NTDs 2021–2030, which emphasises integrated approaches combining chemotherapy, improved water and sanitation, vector control and strengthened surveillance systems [6].

### 1.2. Biology and Transmission

Human schistosomiasis is caused by blood flukes of the genus *Schistosoma*, which comprises more than 130 species; five species cause most human disease: *S. haematobium*, *S. mansoni*, *S. japonicum*, *S. mekongi*, and *S. intercalatum* [7,8]. *S. haematobium* causes urogenital disease, while the other listed species cause intestinal schistosomiasis.

Schistosomes have an indirect life cycle involving a definitive host (humans, cattle, or sheep) and an intermediate host (freshwater snails—*Bulinus* spp. for *S. haematobium* and *Biomphalaria* spp. for *S. mansoni*) [9]. Transmission occurs through contact with freshwater contaminated by cercariae released from infected snails. Environmental and climatic factors strongly influence transmission dynamics, and climate change may expand suitable snail habitats, altering endemicity and complicating control strategies [10].

Emerging hybrid species add another layer of complexity. Notably, a cluster of Belgian travellers infected in SA in 2016–2017 with a *S. mattheei × S. haematobium* hybrid confirmed the presence of zoonotic hybrids locally, raising concerns for surveillance, diagnosis, and control [11].

### 1.3. Clinical Impact

The clinical spectrum ranges from acute manifestations (swimmer’s itch, fever, diarrhoea, lymphadenopathy) to chronic disease with severe systemic complications, including hepatic fibrosis, portal hypertension, bladder damage, kidney failure, anaemia, growth impairment in children, and increased risk of bladder cancer [2].

Urogenital schistosomiasis, primarily caused by *S. haematobium*, damages the bladder, ureters, and reproductive organs. Coined ’a neglected disease within a neglected disease‘, female genital schistosomiasis (FGS) is an under-recognised but highly consequential manifestation, affecting an estimated 56 million women and girls globally, mostly in sub-Saharan Africa [12]. FGS increases the risk of HIV acquisition up to three-fold, causes infertility, pregnancy complications, and chronic gynaecological morbidity, and is often misdiagnosed as a sexually transmitted infection, underdiagnosed and inadequately addressed in reproductive health services [1,12]. A study in KwaZulu-Natal, South Africa, estimates FGS prevalence among adolescent girls around 46%, with praziquantel treatment showing potential benefit, though major gaps remain in health system integration [13]. Male genital schistosomiasis (MGS) affects the prostate, seminal vesicles, epididymis, and testes, leading to haematospermia, prostatitis, infertility, and tumour-like masses [14]. While more data are available from Nigeria, Madagascar, Egypt and Zimbabwe, South African evidence remains sparse [14].

### 1.4. Diagnosis and Treatment

Diagnostic tools include microscopy, antigen detection, antibody assays, and nucleic acid amplification tests (NAATs), each with varying sensitivity and specificity [15]. Despite research progress, no vaccine is available. Praziquantel, in use since the 1980s, remains the cornerstone of treatment and mass drug administration (MDA) campaigns [2,8,9,16].

### 1.5. South African Situation

In South Africa, schistosomiasis is considered moderately endemic (10–50% prevalence) [1]. Transmission is concentrated in Limpopo, Mpumalanga, northern/eastern Gauteng, low-altitude KwaZulu-Natal, and parts of the Eastern Cape [1].

Historical prevalence studies, including school surveys, confirmed high endemicity [17]. A study in Ehlanzeni District, Mpumalanga, found a striking 75% prevalence [18]. Previous national laboratory-based analyses reported a crude case detection rate of approximately 36 per 100,000 population between 2011 and 2018 [17]. However, true prevalence remains uncertain due to reliance on passive surveillance systems.

### 1.6. Policy and Control Framework

The WHO released updated schistosomiasis control and elimination guidelines in February 2022, recommending test-and-treat, preventive chemotherapy, and expanded MDA as critical components. South Africa’s Essential Medicines List includes praziquantel, but broader public health implementation has been inconsistent.

The South African NTD Master Plan prioritises schistosomiasis control, and the new MDA measures are in accordance with WHO goals. Surveillance gaps (including NMC data capture), under-recognised manifestations (FGS/MGS), and weak integration into reproductive and HIV care remain significant barriers.

Strengthened data systems, integrated reproductive health services and robust surveillance will be essential for guiding policy. Evidence from laboratory-confirmed cases, prevalence studies, and pilot MDA programmes should support a national control and elimination strategy, advancing SA towards the 2030 WHO NTD targets.

Routine laboratory data provide an important opportunity to monitor trends at a national scale, particularly in settings where population-based surveys are resource-intensive. However, these data have important limitations, including healthcare access marked by deep disparities shaped by structural, economic, policy, and socio-cultural factors and underdetection.

This study aims to describe national trends in laboratory-confirmed schistosomiasis in South Africa between 2019 and 2024 and to assess how differences in diagnostic intensity influence the observed burden.

## 2. Materials and Methods

### 2.1. Data Source and Population

This retrospective descriptive study was undertaken to investigate trends in the rate of laboratory-confirmed cases of schistosomiasis in South Africa between 1 January 2019 and 31 December 2024; it builds on and refines the earlier analysis of De Boni et al. (2011–2018) [17]. The study population was those who utilise public sector health care and routine diagnostic laboratory services provided by the National Health Laboratory Service (NHLS) across all nine provinces of South Africa. A centralised repository (Surveillance Data Warehouse, SDW) at the National Institute for Communicable Diseases (NICD), NHLS, accumulates laboratory result data from the NHLS and other sources. The primary data source was results of microscopy tests conducted for schistosomiasis stored in the SDW. Two data extracts were performed, one to determine the total volume of schistosomiasis test requests and a second to determine the number of confirmed positive tests.

### 2.2. Case Definition

Confirmed schistosomiasis was defined as the microscopic detection of *S. haematobium* and/or *S. mansoni* eggs in urine, stool or other appropriate specimens.

### 2.3. Inclusion and Exclusion Criteria

As *S. japonicum* is an exotic species to South Africa, reports of this species were excluded from analysis. *S. haematobium* infection may be indicated by the presence of haematuria. However, as this is not a conclusive diagnosis, individuals who tested positive for haematuria but had no ova observed on urine microscopy for schistosomiasis were excluded. Laboratory results captured for proficiency testing purposes and duplicate records were excluded.

Repeated positive results within 60 days were considered part of the same infection episode and excluded. Positive results ≥60 days were classified as reinfections and included.

Serological results were excluded due to the inability to distinguish active from past infection.

### 2.4. Data Management and Cleaning

**Figure 1:**
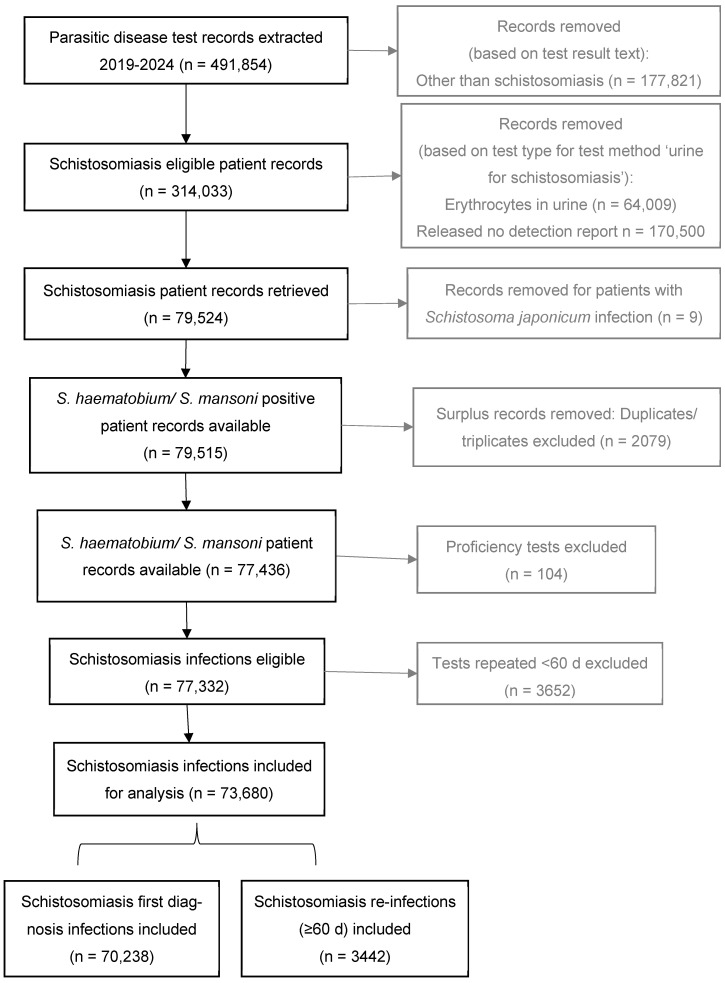
Data extraction process and inclusion/exclusion criteria of public NHLS laboratory data, South Africa, 2019–2024.

Data were imported into Stata^®^ 18.0 [Stata Corporation, College Station, TX, USA] for data deduplication, further refinement, coding, and analysis (Figure 1). In order to deduplicate the set of test records in Stata 18.0, we employed a deterministic linking method that relies on exact matches using unique patient identifiers and variables, such as non-missing date of birth, test date, and laboratory name.

Based on qualifying inclusion values in test result variables, a binary variable for the outcome—the absence or presence of *Schistosoma* spp.—was created. Variable year was created from date variables. The missing age values were replaced by calculated values from dates of birth. The province was that of the facility where the patient presented.

In Stata 18.0, record observations were conditionally dropped according to the criteria by vectorizing logistical operations on variables. A variable that classified records as first diagnosis, re-infection and repeated tests based on a 60 days’ interval between urine/stool collection dates was created, and the repeated test records occurring within 60 days of the first one were dropped from the dataset.

### 2.5. Data Analysis

The age-specific rates and cumulative rates of schistosomiasis test positivity by biological sex and per province were calculated per 100,000, using South Africa’s mid-year population estimated from the 2022 census by Statistics SA as the denominator. Temporal trends were analysed using the Joinpoint regression software version 4.9.1.0. (Statistical Research and Applications Branch, National Cancer Institute, Bethesda, MD, USA). Joinpoint regression analysis is an analytical instrument that is used to investigate trends in sequential data. It identifies critical junctures to divide the data into phases, applies linear or log-linear models to each phase, and analyses the unique variation characteristics within each phase over the course of the entire duration. The trend data, i.e., the positivity rates (PR), were fitted to the simplest Joinpoint model that the data allowed [19]. This determined if an apparent change in trend, namely average annual percent change (AAPC), was statistically significant for the reporting period. The tests of significance used a Monte Carlo Permutation method. The model assumed that the slope changes significantly at the joinpoint, which can be statistically significant, and there were minimum requirements of two data points before and after the joinpoint to generate segments with stronger confidence. Graphs and maps were generated using the Joinpoint software, Microsoft Excel 2016 and ArcGIS Pro 2.3.0 (Redlands, CA, USA) to show the various annual and cumulative trends and relationships in the data.

Due to the non-normal distribution of data, the log-linear model for trend analysis was used. The response variable was the natural logarithm of the annual infection rates per population of 100,000 and the independent variable was the year. In a second simulation, the response variable was cumulative infection rates per age group per 100,000 population and the independent variable was sex.

Test positivity rates were also adjusted using a test rate ratio (TRR) to correct for the patient test volumes at each site, in this way appreciating the difference in facilities testing and volumes and cases between the provinces and TRR can be interpreted as any rate ratio. The TRR formula is as follows:

TRR=test count (reference group)population (reference group)test count (comparison group)population (comparison group)

The reference group was chosen based on the data (aggregated by province) that showed the highest period positivity rate. The adjusted positivity rate (APR) was calculated as the product of the PR and TRR:

APR=PR×TRR

Thus, if the TRR is more than 1.0, it indicates that the comparison group underwent less testing (relative to population size) than the reference group, and the effect was multiplicative. Test rate ratios help distinguish whether observed differences in case numbers may reflect the true disease burden or differences in healthcare access and testing practices.

We also calculated the percentage of all microscopy schistosomiasis tests performed that were positive:

$$PP=\frac{positive\;tests}{total\;tests}\times 100\%$$

## 3. Results

### 3.1. Description of Schistosomiasis Cases

Overall, 73,680 schistosomiasis cases were microscopically diagnosed in the public health sector in South Africa between 2019 and 2024; the median annual number of cases was 11,933, IQR: 11,221–12,910 (median, interquartile range).

In 73,598 or 99.9% of the cases (99.9% with urine samples tested), *S. haematobium* was identified, whereas less than 1.1 percent of the cases (98.8% with stools tested) were infected with *S. mansoni*. Most tests (90%) were performed on urine samples, 9.3% on stool samples, and a small number of other specimens were tested: swabs (*n* = 1), aspirates (*n* = 3), tissue (*n* = 3) and sputum (*n* = 1). The laboratory test volumes, in descending order by province, were: Gauteng (40.2%), KwaZulu-Natal (17.4%), Western Cape (16.8%), and Eastern Cape (8.1%). Each of the remaining provinces contributed 5% or less: Free State (5.0%), Mpumalanga (4.5%), Limpopo (4.0%), North West (2.9%), and Northern Cape (1.3%).

**Figure 2:**
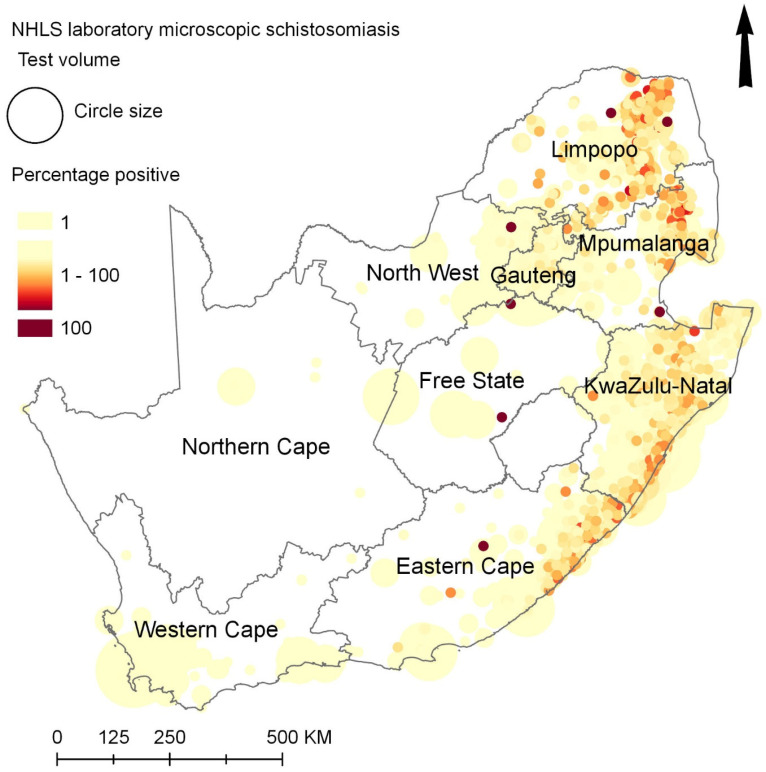
Microscopic schistosomiasis test volumes (circle size) and percentage positivity (circle colour) by public sector NHLS laboratories in South Africa, 2019–2024 (https://dataportal-mdb-sa.opendata.arcgis.com/, accessed on 4 March 2025).

Cases were geographically concentrated in the north-eastern part of the country, with 89.5% of the schistosomiasis cases originating from three provinces, i.e., KwaZulu-Natal, Limpopo and Mpumalanga (Figure 2). The Eastern Cape Province added another 7.7% to the total cases, while the Gauteng Province accounted for 1.6% of cases. Outside the endemic area, the Western Cape Province detected 1.0% of cases, and the remaining three provinces had few cases, accounting for 0.2%. The highest percentage positive (PP) was detected in the Limpopo (9.9%), Mpumalanga (4.9%), KwaZulu-Natal (3.0%), and Eastern Cape provinces (1.2%); in the remaining five provinces, the detection rate was below 1 percent (see Table 1). The laboratory facilities’ test volumes and PPs are displayed in Figure 2.

The PP was highest in the 10–14-year-old children (19.3%), followed by the 5–9-year age group (7.6%) and then the 15–19-year-olds (5.7%). The cumulative age group (5–19 years) accounted for more than half, or 80.5%, of the schistosomiasis cases, and 94.9% of the cases were between 5 and 39 years of age. The PP was 2.8% in males compared to female cases (0.5%), with the sex ratio of cases being 32 females per 100 males.

### 3.2. Schistosomiasis Test Positivity Rate Trends in 2019–2024

The annual crude positivity rate of schistosomiasis in South Africa over the studied 6-year period was 20, IQR: 18–21 (median, interquartile range) and 20, R: 17–25 (mean, range) per 100,000 population.

**Figure 3:**
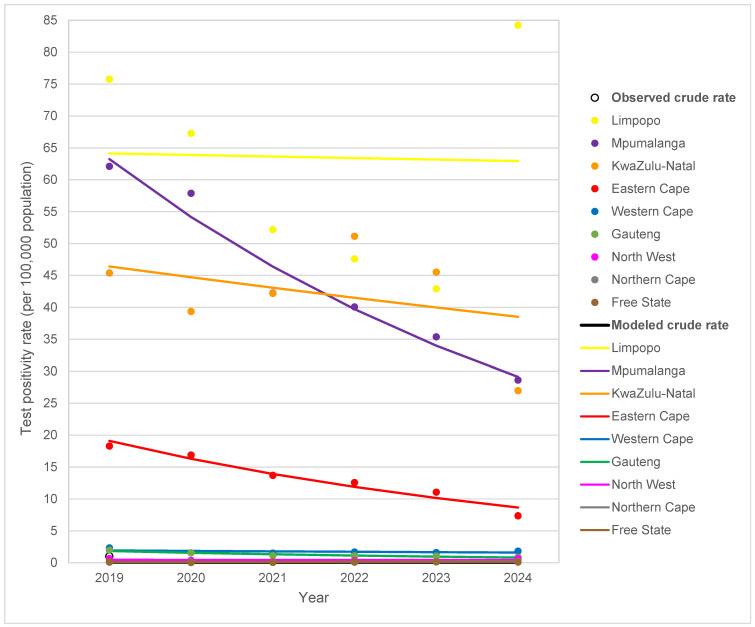
Microscopically diagnosed schistosomiasis crude test positivity rate (observed (point) and modelled (line)), by province in South Africa by year, 2019–2024.

Provincial trends varied (Figure 3) with Limpopo Province showing the highest crude rate of schistosomiasis throughout the reporting period, with a median annual rate of 59.7 cases per 100,000 population. Mpumalanga and KwaZulu-Natal provinces follow, with higher rates in the former province in the first half and the latter province in the second half of the reporting period. Their respective median annual rates were 41.2 and 43.8 cases per 100,000 population. Next was the Eastern Cape Province with a median annual rate of 13.1 cases per 100,000 population. The Western Cape and Gauteng provinces had median annual values between 1 and 2 cases per 100,000 population, respectively, and the remaining provinces (North West, Free State, and Northern Cape) had below 1 case per 100,000 population.

In Figure 3 and Table 2, a downward trend in crude test positivity of schistosomiasis is observed in Mpumalanga, Eastern Cape, and Gauteng provinces, with significant negative APC values (*p* < 0.000001). The Limpopo’s modelled crude rate trend was stable; however, the observed crude rate spiked in the province in 2024 (Figure 3) and more specifically in September and October of that year (Figure S1).

**Figure 4:**
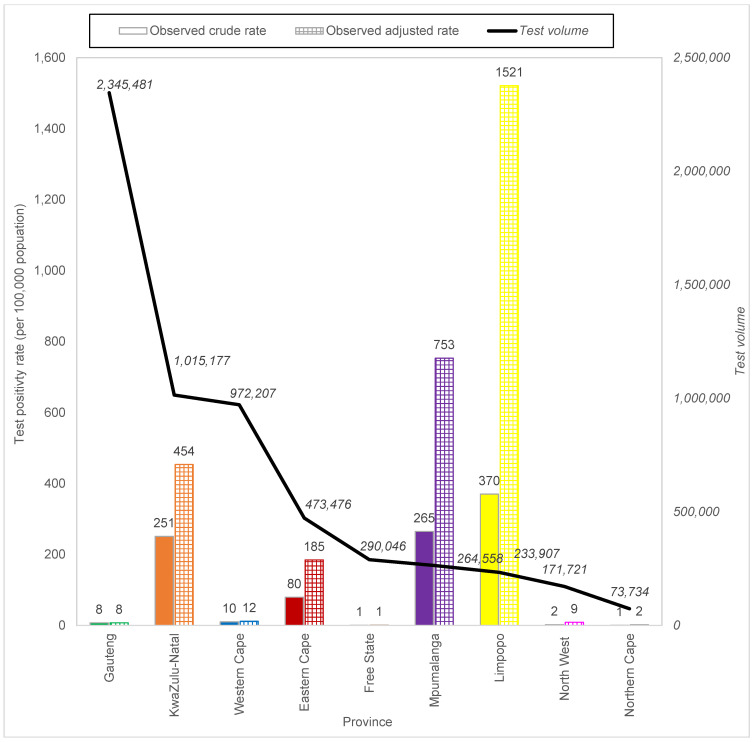
Microscopically diagnosed schistosomiasis observed crude and adjusted test positivity rates (bar chart primary *x*-axis) and test volume (line secondary x-axis) by province in South Africa by year, 2019–2024.

In Figure 4, for the provinces with the highest crude test positivity rates, it is shown that the Limpopo and Mpumalanga provinces have relatively very low test volumes compared to Gauteng Province, chosen as a reference for the test rate ratio calculation of the other provinces. As a result, the crude and adjusted rates of schistosomiasis differ greatly because of the substantial adjustment made. The crude rates for the KwaZulu-Natal and Eastern Cape provinces were also adjusted for differences in test volumes, but the effects were lower because of relatively higher test volumes than in the other provinces, and still much lower than those in Gauteng province. A trend analysis performed on the adjusted rates (dependent variable) using province as a co-variable and year as an independent variable did not differ much from the crude rate analysis in Figure 3 and Table 2 and is, for that reason, not shown.

**Figure 5:**
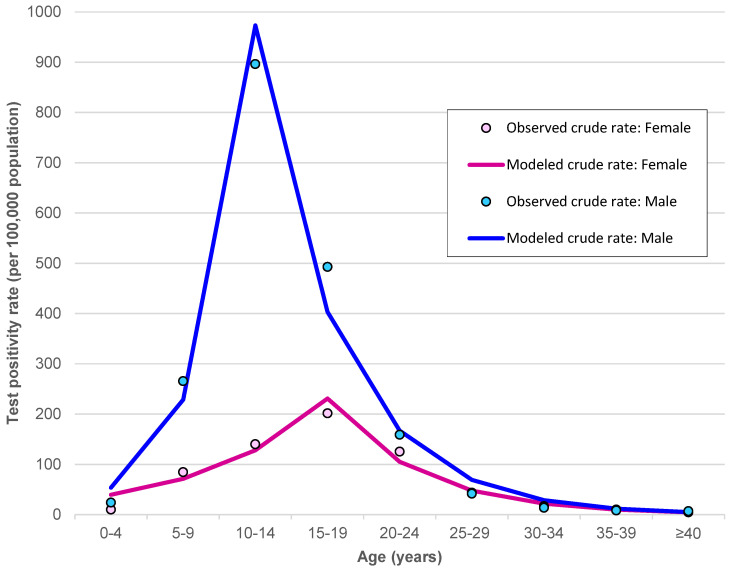
Microscopically diagnosed schistosomiasis crude test positivity rate (observed (point) and modelled (line)) by sex and age in South Africa by year, 2019–2024.

The median values, 41.7 cases per 100,000 population for males and 43.6 cases per 100,000 population for females, were not different. Despite this, Figure 5 shows that test positivity rates were vastly different between younger and older ages. For males, the 10–14-year age group had the highest crude rate of 896.4 cases per 100,000 population. For females, the 15–19-year age group had the highest crude rate of 201.6 cases per population of 100,000. The peak was much more pronounced with males (Figure 5), but values were similar between the sexes from age 25 years upwards. The joinpoints are also located on the 10–14 and 15–19-year age groups for males and females, respectively (Table 3). The trends were significantly upward, going to the joinpoints and significantly downward when moving beyond the joinpoints, as indicated by the respective positive and negative slopes, percentage change between age groups, and *p*-values of <0.000001 (Table 3). The difference between slopes was negative, i.e., the downward trend was across a higher number of age groups, and the upward trend with high values (specifically for males) was over 2–3 age groups only (Figure 5 and Table 4). 

**Table 3:** Microscopically diagnosed schistosomiasis age group percentage change (APC) in crude test positivity rate trends by sex in South Africa, 2019–2024.

Crude Cohort	Segment **	Lower Endpoint	Upper Endpoint	APC	Lower CI	Upper CI	*p*-Value
Female	1	0	3	80.2656 *	38.6324	153.6163	<0.000001
Female	2	3	8	−54.5619 *	−66.1000	−49.3450	<0.000001
Male	1	0	2	325.5392 *	195.1648	900.7273	0.000400
Male	2	2	8	−58.5859 *	−72.1317	−53.5132	<0.000001

* Indicates that the age group percentage change (APC) is significantly different from zero at alpha-level = 0.05. A test statistic is not available for the empirical quantile method. ** 1 Joinpoint (2 segments).

**Table 4:** Estimated regression coefficients (beta), standardised parametrization of microscopically diagnosed schistosomiasis trends by age group and sex in South Africa, 2019–2024.

Cohort	Parameter	Parameter Estimate	Standard Error	Test Statistic (t)	Prob > |t|
Female—1 Joinpoint	Intercept 1	3.674407	0.420693	8.734180	0.000947
Female—1 Joinpoint	Slope 1	0.589261	0.253454	2.324923	0.080700
Female—1 Joinpoint	Slope 2—Slope 1	−1.378081	0.284838	−4.838120	0.008412
Male—1 Joinpoint	Intercept 1	3.984278	0.498997	7.984568	0.001334
Male—1 Joinpoint	Slope 1	1.448187	0.521413	2.777427	0.049950
Male—1 Joinpoint	Slope 2—Slope 1	−2.329736	0.528697	−4.406560	0.011632

## 4. Discussion

This national analysis of laboratory-confirmed schistosomiasis cases in South Africa between 2019 and 2024 provides an updated perspective on the demographic distribution, geographic concentration, and temporal trends of infection across the country. Using routine public-sector laboratory surveillance data, the study confirms that schistosomiasis remains an important yet geographically focal public health problem, disproportionately affecting children and adolescents in endemic provinces.

### 4.1. Burden and Geographic Distribution

The vast majority of laboratory-confirmed schistosomiasis cases originated from three endemic provinces—KwaZulu-Natal, Limpopo, and Mpumalanga—accounting for 89.5% of all detected cases. This spatial concentration reaffirms prior studies indicating that north-eastern South Africa remains the hotspot for transmission. Limpopo Province had the highest positivity rate (9.9%), despite relatively lower test volumes, indicating high local endemicity. Schistosomiasis transmission does not occur in the Western Cape Province because of environmental unsuitability for snail hosts. Cases diagnosed in this province acquired the infection elsewhere, probably mainly in the Eastern Cape Province. Although parts of Gauteng Province are susceptible to transmission, the majority of cases recorded in this province are believed to be migrants from highly endemic areas. Most cases detected in these provinces likely represent infections acquired elsewhere, reflecting internal migration patterns and healthcare utilisation in metropolitan referral centres.

Adjustment of positivity rate using test rate ratios demonstrated that crude rates may underestimate burden in provinces with lower diagnostic volumes. These findings emphasise that surveillance indicators must account for diagnostic access disparities when interpreting regional disease patterns.

### 4.2. Comparison with Previous National Surveillance

This study extends national laboratory-based surveillance findings from 2011 to 2018, which reported a higher crude prevalence of 36 cases per 100,000 population [17]. The reduction observed in the present analysis to approximately 20 per 100,000 population may reflect improved deduplication methods, changing healthcare utilisation patterns, or early impacts of control interventions rather than true reductions in transmission. Passive laboratory surveillance captures only individuals presenting for care and undergoing testing, and therefore likely underestimates true prevalence, particularly in rural communities.

However, the persistence of similar demographic and geographic patterns across both surveillance periods supports the reliability of routine laboratory data as an important monitoring mechanism. Continued surveillance will be critical for assessing the future programme impact as national mass drug administration (MDA) of praziquantel initiatives expand.

### 4.3. Age and Sex Patterns

Children and adolescents bore the highest burden, with the 10–14-year-old group showing the highest test positivity (19.3%), followed by the 5–9 and 15–19 age groups. Notably, the cumulative group of 5–19 years accounted for over 80% of cases. This age distribution aligns with known exposure risks due to water-based recreational and domestic activities that are common among school-age children [1].

Males were disproportionately affected, representing nearly 73% of cases and showing significantly higher positivity (2.8%) compared to females (0.5%). This sex disparity is well-documented and may relate to behavioural differences in water exposure. Interestingly, the sex ratio equalised above 25 years, reflecting a convergence of risk with ageing.

Reliance solely on microscopy likely underestimates FGS-related morbidity among adolescent girls and women, which often presents without detectable egg excretion in urine microscopy [12,20]. Integration of schistosomiasis screening into sexual and reproductive health services may therefore improve detection and management.

### 4.4. Temporal Trends

The national average test positivity rate remained relatively stable (median 20 per 100,000), but significant geographic trends emerged. The Eastern Cape, Gauteng, and Mpumalanga provinces showed statistically significant declines in rate over the study period. In the absence of implementation of any large-scale treatment and control efforts in these provinces, the most likely explanation for the apparent decline is the negative effect of COVID-19 on healthcare access.

Limpopo Province maintained persistently high rates suggests continued transmission requiring strengthened interventions. In 2024, increased case detection led to a substantial rise in test volumes and positive results, driven by expanded testing efforts and improved detection among children of school-going age (Supplementary Information). KwaZulu-Natal Province’s stable rate, despite substantial test volumes, may reflect improved detection or early programme impacts that are yet to show a measurable reduction Specifically, MDA of praziquantel is yet to be fully implemented, following a limited pilot phase in 2024.

Age-specific trend analysis revealed significant increases in test positivity rates up to a peak age (10–14 years for males, 15–19 years for females), followed by sharp declines. The peak was markedly higher among males (896.42/100,000), supporting the need for targeted interventions for adolescent boys.

The initiation of MDA pilots in KwaZulu-Natal in late 2024 and anticipated national scale-up from 2026 offer an important opportunity to use laboratory surveillance as a baseline against which programme effectiveness can be measured.

### 4.5. Diagnostic and Surveillance Considerations

Microscopy of urine and stool remains the routine diagnostic standard in South Africa but has limited sensitivity, particularly in low-intensity infections. The overwhelming predominance of *Schistosoma haematobium* reflects the urogenital dominance of disease locally. However, sporadic detection of *S. mansoni* and emerging reports of hybrid schistosomes elsewhere in Africa highlight the need for continued molecular surveillance capacity at reference laboratories [21].

Routine surveillance systems currently capture only classical urinary or intestinal disease, while genital and chronic manifestations remain largely invisible within laboratory reporting systems. Incorporating histopathology-based tissue diagnoses into national surveillance frameworks would substantially improve detection of chronic and ectopic schistosomiasis, including female and male genital schistosomiasis, hepatointestinal disease, and tumour-associated schistosomal pathology that often present outside routine urine and stool microscopy pathways. Histological examination frequently identifies schistosome ova incidentally in biopsies and surgical specimens from the bladder, cervix, prostate, fallopian tubes, liver, and colorectal tissues, representing missed opportunities for case notification and burden estimation. Integration of pathology laboratory data, including digital pathology platforms and structured reporting of schistosomiasis-associated lesions, into surveillance systems would therefore enable improved morbidity mapping, strengthen programme impact assessment, and better quantify long-term complications such as infertility and schistosomiasis-associated bladder and cervical pathology. Such integration would also support earlier clinical recognition and treatment of chronic disease manifestations, improving both patient outcomes and national disease burden estimation.

Integrating schistosomiasis into reproductive health and HIV services could substantially improve burden estimation and patient care.

### 4.6. Public Health Implications

These findings reinforce that schistosomiasis control in South Africa should remain focused on school-aged children and adolescents, high-burden endemic provinces, improved diagnostic access in rural districts, integration into primary healthcare and reproductive health services, and continued expansion of MDA programmes.

Long-term control will depend not only on chemotherapy but also on improvements in water infrastructure, sanitation, health education, and environmental management.

Lower testing rates in high-prevalence provinces such as Limpopo and Mpumalanga highlight the need to strengthen diagnostic capacity and surveillance systems, to improve case detection and reporting. Enhanced provincial coordination and integration with the WHO roadmap strategies are essential to achieve elimination targets.

Routine laboratory surveillance offers a scalable and sustainable mechanism to monitor progress towards the WHO NTD elimination targets but requires strengthened reporting systems and provincial utilisation for decision-making.

### 4.7. Study Limitations

This analysis is limited to individuals accessing public healthcare and undergoing laboratory testing. Asymptomatic infections, private-sector diagnoses, and subclinical genital disease are not captured. Microscopy sensitivity limitations and uneven testing practices may underestimate the disease burden. Furthermore, the exclusion of adjunctive diagnostic markers such as serology and haematuria testing likely resulted in additional under-detection of cases. Population mobility and migration patterns further complicate the attribution of infection to specific provinces.

Despite these limitations, routine laboratory surveillance remains one of the most practical national monitoring systems available and provides critical insights for programme planning.

### 4.8. Future Directions

Future work should aim to link laboratory surveillance with programme implementation metrics, incorporate FGS and MGS surveillance indicators, expand molecular diagnostics for hybrid species detection, develop real-time provincial surveillance dashboards, and combine routine data with targeted community prevalence surveys.

Such efforts will strengthen evidence-based schistosomiasis control strategies in South Africa.

## 5. Conclusions

This six-year national laboratory-based analysis confirms that schistosomiasis remains a geographically focal yet persistent public health concern in South Africa. Children and adolescents aged 5–19 years, particularly males in the KwaZulu-Natal, Limpopo, and Mpumalanga provinces, continue to bear the greatest burden of disease. While apparently declining trends in some provinces offer cautious optimism, sustained transmission persists in key endemic regions and significant gaps in testing and reporting remain. Ongoing and expanded MDA efforts, especially among school-aged children in endemic provinces, are critical. Strengthening laboratory surveillance, integration of schistosomiasis management into primary and reproductive healthcare services, enhancing awareness among the public and healthcare workers, and improving access to praziquantel treatment will be key pillars in supporting national and global schistosomiasis control goals by 2030.

Routine laboratory surveillance provides a scalable mechanism for monitoring schistosomiasis burden in middle-income countries where nationwide prevalence surveys remain costly and logistically challenging. This study demonstrates how routine diagnostic data can inform targeted interventions, optimise resource allocation, and establish baseline trends for evaluating MDA impact. Strengthening surveillance integration with public health programmes will be critical for sustainable disease control. This approach aligns with recent advances in neglected tropical diseases (NTDs), which emphasise data-driven strategies, integrated control programmes, and the use of routine health information systems to accelerate progress towards the elimination targets [6].

## Figures and Tables

**Table 1:** Microscopically diagnosed schistosomiasis cases, tests numbers, proportion of cases and tests by sex, age, and province in public healthcare in South Africa, 2019–2024.

Characteristic	Number of Cases	Proportion of Cases	Number of Tests	Proportion of Positive Tests
Sex				
Female	17,258	23.42%	3,824,614	0.45%
Male	53,578	72.72%	1,901,686	2.82%
Not specified	2844	3.86%	114,009	2.49%
Age				
0–4	1035	1.40%	417,945	0.25%
5–9	10,521	14.28%	137,774	7.64%
10–14	30,950	42.01%	160,302	19.31%
15–19	17,813	24.20%	315,795	5.65%
20–24	6924	9.40%	540,335	1.28%
25–29	2384	3.19%	636,304	0.37%
30–34	895	1.21%	611,009	0.15%
35–39	478	0.65%	506,521	0.09%
40+	1029	1.40%	1,863,147	0.06%
Not specified	1669	2.27%	651,177	0.26%
Province				
Eastern Cape	5683	7.71%	473,476	1.20%
Free State	19	0.03%	290,046	0.01%
Gauteng	1157	1.57%	2,345,481	0.05%
KwaZulu-Natal	29,938	40.63%	1,015,177	2.95%
Limpopo	23,094	31.34%	233,907	9.87%
Mpumalanga	12,924	17.54%	264,558	4.89%
North West	95	0.13%	171,721	0.06%
Northern Cape	10	0.01%	73,734	0.01%
Western Cape	760	1.03%	972,207	0.08%
Not specified	0	0	2	
South Africa	73,680	100.00%	5,840,309	1.26%

**Table 2:** Microscopically diagnosed schistosomiasis annual percentage change (APC) in crude test positivity trends by province in South Africa, 2019–2024.

Crude Cohort	Lower Endpoint	Upper Endpoint	APC	Lower CI	Upper CI	*p*-Value
Eastern Cape	2019	2024	−14.6248 *	−20.1977	−9.7201	<0.000001
Free State	2019	2024	−3.7871	−30.6300	38.2132	0.751050
Gauteng	2019	2024	−14.8811 *	−20.6932	−9.7205	<0.000001
Kwazulu-Natal	2019	2024	−3.6611	−18.2071	13.4396	0.551090
Limpopo	2019	2024	−0.3779	−23.5193	28.2802	0.961808
Mpumalanga	2019	2024	−14.3635 *	−17.7001	−11.5896	<0.000001
North West	2019	2024	−0.7299	−39.5109	60.3888	0.944611
Northern Cape	2019	2024	20.5520	−15.8783	97.7025	0.214357
Western Cape	2019	2024	−3.6563	−18.0114	13.0702	0.494301

* Indicates that the annual percentage change (APC) is significantly different from zero at alpha-level = 0.05. A test statistic is not available for empirical quantile method.

## Data Availability

The raw data that substantiate the findings of this study are not publicly accessible due to sensitivity concerns, despite their de-identification. Even so, the authors are able to provide aggregated data upon reasonable request and with the permission of the Academic Affairs Research and Management System (AARMS) of the NHLS.

## Supplementary Materials

The following supporting information can be downloaded at: https://www.mdpi.com/article/10.3390/tropicalmed11060154/s1, see Figure S1: Microscopically diagnosed schistosomiasis cases in Limpopo province, 2019–2024.

## References

[1] Schistosomiasis (Bilharzia). https://www.who.int/health-topics/schistosomiasis.

[2] Colley D.G., Bustinduy A.L., Secor W.E., King C.H. (2014). Human Schistosomiasis. Lancet.

[3] van der Werf M.J., de Vlas S.J., Brooker S., Looman C.W.N., Nagelkerke N.J.D., Habbema J.D.F., Engels D. (2003). Quantification of Clinical Morbidity Associated with Schistosome Infection in Sub-Saharan Africa. Acta Trop..

[4] Magaisa K., Taylor M., Kjetland E.F., Naidoo P.J. (2015). A Review of the Control of Schistosomiasis in South Africa. S. Afr. J. Sci..

[5] Fenwick A., Webster J.P., Bosque-Oliva E., Blair L., Fleming F.M., Zhang Y., Garba A., Stothard J.R., Gabrielli A.F., Clements A.C.A. (2009). The Schistosomiasis Control Initiative (SCI): Rationale, Development and Implementation from 2002–2008. Parasitology.

[6] World Health Organization (2020). Ending the Neglect to Attain the Sustainable Development Goals: A Road Map for Neglected Tropical Diseases 2021–2030.

[7] Vere M., ten Ham-Baloyi W., Melariri P.E. (2024). Effects of Paediatric Schistosomiasis Control Programmes in Sub-Saharan Africa: A Systematic Review. PLoS ONE.

[8] Mutapi F., Maizels R., Fenwick A., Woolhouse M. (2017). Human Schistosomiasis in the Post Mass Drug Administration Era. Lancet Infect. Dis..

[9] McManus D.P., Dunne D.W., Sacko M., Utzinger J., Vennervald B.J., Zhou X.-N. (2018). Schistosomiasis. Nat. Rev. Dis. Primers.

[10] McCreesh N., Nikulin G., Booth M. (2015). Predicting the Effects of Climate Change on *Schistosoma mansoni* Transmission in Eastern Africa. Parasites Vectors.

[11] Cnops L., Huyse T., Maniewski U., Soentjens P., Bottieau E., Van Esbroeck M., Clerinx J. (2021). Acute Schistosomiasis with a *Schistosoma mattheei* × *Schistosoma haematobium* Hybrid Species in a Cluster of 34 Travelers Infected in South Africa. Clin. Infect. Dis..

[12] Kjetland E.F., Leutscher P.D.C., Ndhlovu P.D. (2012). A Review of Female Genital Schistosomiasis. Trends Parasitol..

[13] Nemungadi T.G., Kleppa E., Galappaththi-Arachchige H.N., Pillay P., Gundersen S.G., Vennervald B.J., Ndhlovu P.D., Taylor M., Naidoo S., Kjetland E.F. (2024). Predictors for Participation in Mass-Treatment and Female Genital Schistosomiasis Re-Investigation, and the Effect of Praziquantel Treatment in South African Adolescents. PLoS Negl. Trop. Dis..

[14] Kayuni S., Lampiao F., Makaula P., Juziwelo L., Lacourse E.J., Reinhard-Rupp J., Leutscher P.D.C., Stothard J.R. (2018). A Systematic Review with Epidemiological Update of Male Genital Schistosomiasis (MGS): A Call for Integrated Case Management across the Health System in Sub-Saharan Africa. Parasite Epidemiol. Control..

[15] Hoekstra P.T., van Esbroeck M., de Dood C.J., Corstjens P.L., Cnops L., van Zeijl-van der Ham C.J., Wammes L.J., van Dam G.J., Clerinx J., van Lieshout L. (2021). Early Diagnosis and Follow-up of Acute Schistosomiasis in a Cluster of Infected Belgian Travellers by Detection of Antibodies and Circulating Anodic Antigen (CAA): A Diagnostic Evaluation Study. Travel. Med. Infect. Dis..

[16] Hotez P.J., Fenwick A., Kjetland E.F. (2009). Africa’s 32 Cents Solution for HIV/AIDS. PLoS Negl. Trop. Dis..

[17] De Boni L., Msimang V., De Voux A., Frean J. (2021). Trends in the Prevalence of Microscopically-Confirmed Schistosomiasis in the South African Public Health Sector, 2011–2018. PLoS Negl. Trop. Dis..

[18] Njikho S.L., Quan V.C., Mbonane T.P., Van Wyk R.H. (2023). Evaluating the Prevalence and Risk Factors of Schistosomiasis Amongst School-Aged Children in Low- and Middle-Income Communities: Ehlanzeni District Municipality, South Africa, 2015–2021. Trop. Med. Infect. Dis..

[19] Kim H.J., Fay M.P., Feuer E.J., Midthune D.N. (2000). Permutation Tests for Joinpoint Regression with Applications to Cancer Rates. Stat. Med..

[20] World Health Organization (2015). Female Genital Schistosomiasis: A Pocket Atlas for Clinical Health-Care Professionals.

[21] Leger E., Webster J.P. (2017). Hybridizations within the Genus *Schistosoma*: Implications for Evolution, Epidemiology and Control. Parasitology.

