# Supplementary material for: Persistent Burden of Schistosomiasis in South Africa: A National Laboratory-Based Analysis, 2019–2024
Source: Trop Med Infect Dis. 2026 Jun 5;11(6):154. doi: 10.3390/tropicalmed11060154 (PMC13307819; doi:10.3390/tropicalmed11060154)
Supplement: Supplementary file 1 [file tropicalmed-11-00154-s001.zip › tropicalmed-4322346-supplementary.pdf]

Tropical Medicine and Infectious Disease

MDPI

**Supplementary material**

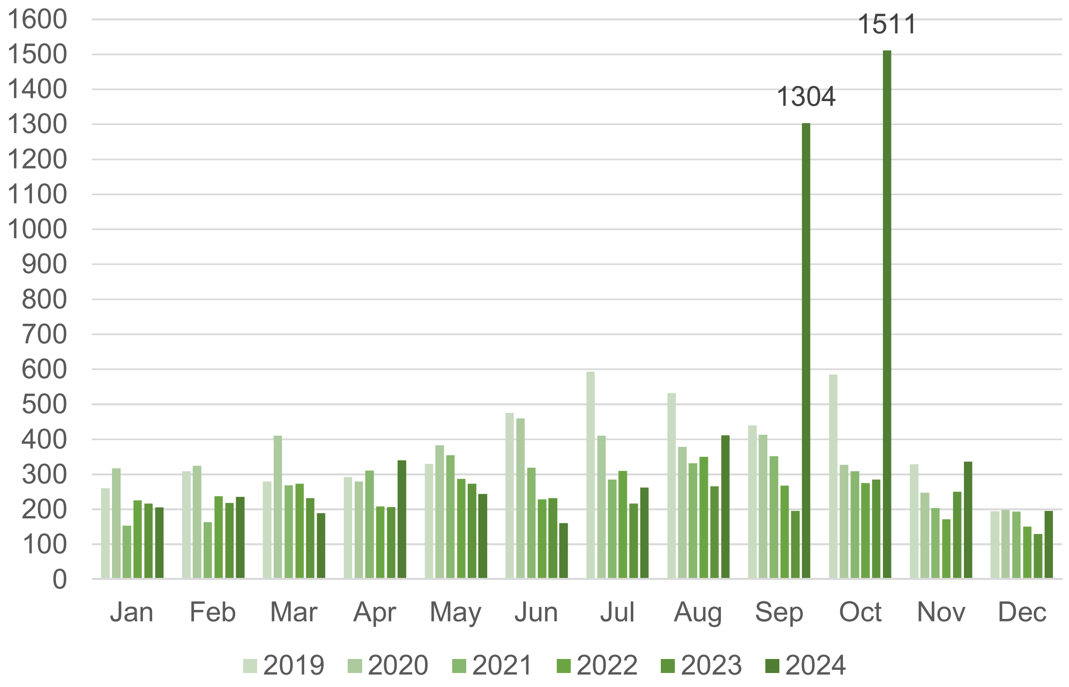


Figure S1: Microscopically diagnosed schistosomiasis cases in Limpopo province, 2019-2024
